# Development and Analysis of Multi-Degree-of-Freedom Piezoelectric Actuator Based on Elephant Trunk Structure

**DOI:** 10.3390/s23146264

**Published:** 2023-07-10

**Authors:** Zheng Li, Kaiwen Wang, Haibo Wang, Xuetong Chen, Xiaoqiang Guo, Hexu Sun

**Affiliations:** 1 School of Electrical Engineering, Yanshan University, Qinhuangdao 066004, China; 2 School of Electrical Engineering, Hebei University of Science and Technology, Shijiazhuang 050018, China; wkw970221@163.com (K.W.); wanghaibo2021102@163.com (H.W.); chenxuetong2020@163.com (X.C.)

**Keywords:** multiple-degrees-of-freedom, bending-longitudinal -bending, no return displacement

## Abstract

In most of the piezoelectric stacked motors studied, the stator usually adopts two compound modes to drive the rotor to do step motion. This design method not only improves the utilization rate of the stator but also improves the torque output to a certain extent and increases the output displacement. In this study, a new type of multi-degree of freedom piezoelectric actuator is proposed for the utilization of a stator. The actuator realizes three compound vibration modes of bending-longitudinal-bending on a single stator, which changes the two compound modes of longitudinal bending and also changes the single motion mode of the stepper motor along a straight line. The rotor is set as a ball to drive it to rotate. The designed motor presents a different driving signal under which the rotor will no longer be accompanied by a return displacement. The finite element method is used to complete the design analysis, and the experimental analysis of the designed motor is carried out after the prototype is made. The multi-degree-of-freedom piezoelectric actuator can achieve a speed of 8.56 mm/c and a driving load of 1200 g at a voltage of 400 v and a working frequency of 42.7 kHz.

## 1. Introduction

In recent years, the development of ultrasonic motors has attracted much attention. On the one hand, it has the characteristics of fast response, no electromagnetic interference, large output torque, and nanoscale resolution. On the other hand, the ultrasonic motor is widely applied in fields such as aerospace, medicine, robot joint, mobile phone camera, and so on [1,2,3,4,5,6,7]. The ultrasonic motor relies on the inverse piezoelectric effect to convert the input electrical energy into mechanical energy of the stator, which drives the rotor to move through friction, and finally into kinetic energy of the rotor.

The basic structure of the piezoelectric driver is mainly composed of the clamping fixed part, the ceramic plate, and the driving foot, in which the displacement amplifying mechanism and the beam structure are included in the driving foot. Based on the connection of these structures, piezoelectric actuators can be divided into series and parallel actuators. Serial-connected type combines these three structures in sequence. For example, Wang et al. proposed a clamping stepper motor. The clamping part of the stepper motor is selected in the middle of two groups of piezoelectric ceramic plates to realize the longitudinal bending compound vibration, which improves the utilization rate of the stator and increases the amplitude to some extent compared with the longitudinal and bending vibration alone [8]. Sandwich sensors such as these are bolted together. It is representative that Liu et al. combined longitudinal and bending vibrations of different orders to achieve two-degree-of-freedom motion in the straight direction. This kind of sandwich motor has only one output contact, and the output trajectory is elliptical akin to that of the general stacking ultrasonic motor [9]. A number of current studies have also proposed various improvement schemes for motor output performance, for example, Wu et al. proposed the use of alumina as a material for the vibrating body, which was utilized in a Langevin-type ultrasonic stator, and the output characteristics of the motor were experimentally demonstrated to be improved [10]. In the literature [11,12,13], the relationship between other parameters such as vibration amplitude and mechanical processing method is revealed by comparing different processing methods from the ultrasonic motor processing perspective. The team still proposed an I-shaped piezoelectric stack motor using longitudinal vibration. The designed motor is different from the previous one, and the driving foot becomes a four-legged drive. Of course, the number of piezoelectric ceramic groups will also increase accordingly. The four driving feet can achieve an elliptical trajectory, so it can achieve a large thrust stroke, but the realization is still a two-degree-of-freedom linear motion [14]. Similarly, a new type of linear piezoelectric actuator with a rectangular structure proposed by Liu’s team also realizes bidirectional linear motion in different stator modes [15]. Wu et al. mixed the operating principles of longitudinal and curved standing waves and applied them to a cross-shaped two-degree-of-freedom linear motor. By adjusting the BSW to adjust the elliptical operation, this design provides the idea for designing a high-power linear motor, but the motion mode is still linear [16]. The driving foot part is the key to contacting the rotor. Reasonable design can increase the amplitude output and reduce the amplitude deviation. Xu et al. designed a compact actuator with high interchangeability, which reduced the influence of assembly error on motor performance to a certain extent. Meanwhile, the comparative analysis of the low-stiffness driving foot and the rigid driving foot found that the low stiffness was more conducive to assembly and the magnification of the flexible driving foot could remain stable under different preloading deformation. This will serve as a reference for future research on motor stability [17]. Zhen et al. studied a single-mode ultrasonic motor. The design adopts an amplitude transformer with an inclined beam. Through the magnification of the amplitude transformer, the longitudinal vibration and bending vibration can reach 3.4 um [18]. Li et al. made a comparative analysis of the driving feet of the stick-slip piezoelectric driver. The three designed driving feet were classified according to their stiffness. The simulation experiment proved that each was suitable for different working conditions, and the designed actuators realized high-speed and stable motion. Liu et al. have analyzed the dynamic model of a strapped linear ultrasonic motor, considering the impact of the clamping part on the motor, and simplifying the contact model of driving the foot into point contact. The proposed dynamic model has been simulated and experimentally analyzed. The model can be applied to other strapped linear ultrasonic motors. Meanwhile, it can be used as a reference for other linear motors [19].

The piezoelectric ultrasonic motor can be divided into resonant and non-resonant according to its vibration state [20,21,22]. The common modes of the resonant motor are longitudinal vibration and bending vibration, and the stepping motion is realized by using the signal with phase difference π/2. Resonant motors can get high-speed operation, but their displacement only stays at the micron level and will be limited. On the contrary, the non-resonant motor thus uses the deformation of the piezoelectric element to directly output the displacement to obtain a higher displacement resolution, while the travel is further increased compared with the resonant motor [23,24]. At present, most of the stepping motion is accompanied by small segments of return displacement, and most of the signals of the ceramic plate are driven by square waves or triangle waves. In this paper, a new type of stacked ultrasonic motor structure is established, and its motion mode under the characteristic signal is analyzed, as its load capacity under resonant frequency, and the no-load speed of the rotor under different frequencies are analyzed.

It can be seen from the above introduction that the current stacked motor mainly focuses on the linear displacement of output, and the driving modes are mostly consistent with two modes (such as longitudinal bending and longitudinal twisting, etc.). The triangular wave signal is often used in the design of stacked motors, which makes the output displacement accompanied by a small segment of return displacement. In terms of output efficiency, compared with the traveling wave ultrasonic motor, the piezoelectric stack ultrasonic machine has lower output displacement efficiency because of the influence of its signal. To solve the above problems, a new ultrasonic motor with three vibration modes is proposed. The innovation points of the proposed motor are as follows:

1. To solve the problem of single freedom of stacked ultrasonic motor, the spherical rotor is taken as the output so that the stacked ultrasonic motor can achieve multiple degrees of freedom; The designed motor adopts three kinds of flexural—longitudinal—bending vibration compound drive, most of the current two modes, using three modes in the stator utilization rate can be improved and beneficial to realize the rotor of multiple degrees of freedom;

2. The return displacement caused by previous triangular wave signals is eliminated, and three different driving signals are designed, all of which are sinusoidal signals that are easy to output.

The rest of this paper is organized as follows. Section 2 introduces the structure and working principle of the elephant trunk ultrasonic motor. In Section 3, the dynamic model of the motor is established, and the geometric analysis of the driving foot is made. In Section 4, the output displacements of each stator along different directions are studied. The load capacity of the motor and the output displacement under different voltage amplitudes are analyzed experimentally. Finally, in Section 5 make a summary of this article.

## 2. Structure and Working Principle of Elephant Trunk Motor with Multiple Degrees of Freedom

### 2.1. Stator and Rotor Structure

**Figure 1:**
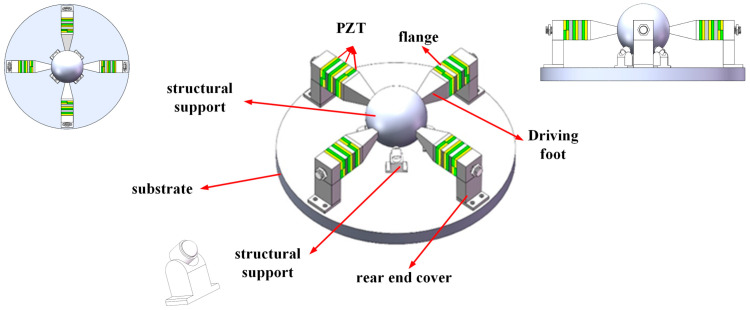
Overall assembly drawing of the Elephant-trunk motor with multiple degrees of freedom.

The assembly drawing of the designed motor is shown in Figure 1. The whole motor is composed of four parts: elephant trunk stator, spherical rotor, disc base, and ball support structure. The four stators are placed symmetrically along the circumferential “X” shape of the ball. The stators are secured to the disc base by screws through circular holes in the bottom rear seat. The rotor adopts a spherical structure to realize multiple degrees of freedom. The support structure holds the rotor at the height of the coaxial line of the stator.

**Figure 2:**
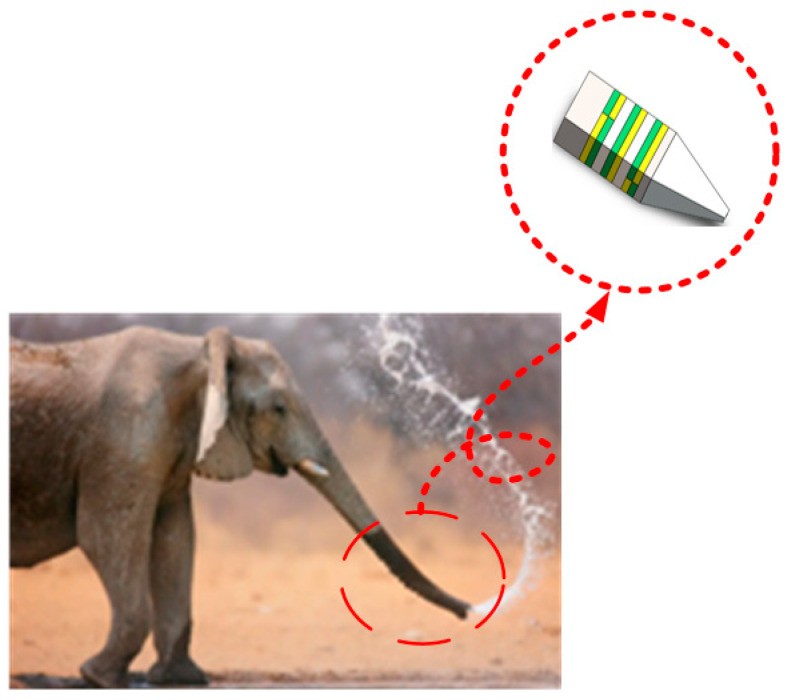
Elephant trunk stator.

The stator shape and working principle of this study are inspired by the elephant trunk, as shown in Figure 2. The trunk can flexibly expand and bend in different directions. The working principle of the piezoelectric stator is flexural vibration—longitudinal vibration—bending vibration.

**Figure 3:**
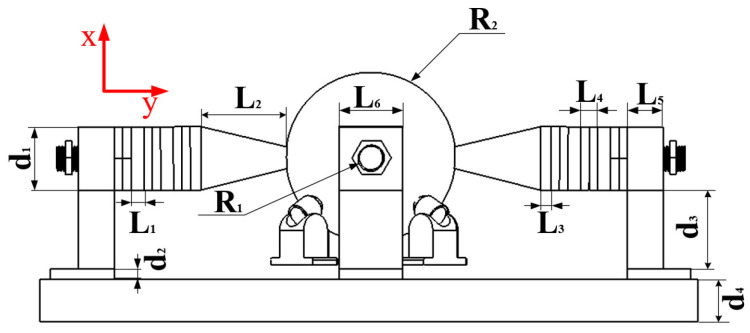
Motor parameter diagram.

The overall size of the designed motor is shown in Figure 3. In order to achieve better frequency degeneracy, the stator structure is transformed into a square. The elephant trunk stator is composed of a rear seat, piezoelectric ceramic plate, flange, and driving foot. Parameters are shown in Table 1 and materials are shown in Table 2.

The steel material density of the base is the highest, which can make the amplitude of the motor concentrate on the stator end as much as possible. Piezoelectric ceramics adopt PZT-5, whose parameters are shown as follows:

(1) $$\varepsilon =\left[\begin{array}{ccc} 8.046 & 0 & 0\\ 0 & 8.046 & 0\\ 0 & 0 & 6.597 \end{array}\right]\times 10^{-9}F/m$$

(2) $$e=\left[\begin{array}{ccc} 0 & 0 & 0\\ 0 & 0 & -4.1\\ 0 & 0 & 14.1\\ 0 & 0 & 0\\ 0 & 10.5 & 0\\ 10.5 & 0 & 0 \end{array}\right]\times 10^{10}c/m^{2}$$

(3) $$C=\left[\begin{array}{cccccc} 13.2 & 7.1 & 7.3 & 0 & 0 & 0\\ 7.1 & 13.2 & 7.3 & 0 & 0 & 0\\ 7.3 & 7.3 & 11.5 & 0 & 0 & 0\\ 0 & 0 & 0 & 3.0 & 0 & 0\\ 0 & 0 & 0 & 0 & 3.0 & 0\\ 0 & 0 & 0 & 0 & 0 & 2.6 \end{array}\right]\times 10^{10}N/m^{2}$$

**Figure 4:**
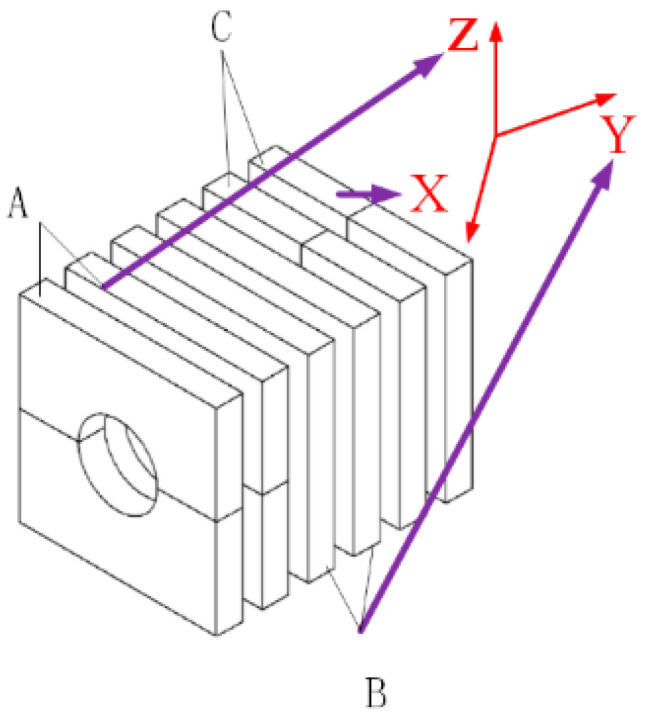
Polarization and arrangement of piezoelectric ceramics.

The rear end seat is fixed on the disc base by a nut, while the rear end fastens each component into a whole through a screw and realizes the application of piezoelectric ceramic pre-pressure. The overall length of the stator is about 70 mm, and the maximum diameter is 30 mm. The overall length larger than the diameter can reduce the inconsistent position of the signal along the axial excitation and avoid uneven amplitude. The piezoelectric piles of the stator are all polarized along the thickness direction and are all treated with circular grooves to obtain higher output torque. As shown in Figure 4, the two adjacent piezoelectric plates have opposite polarization directions. The piezoelectric piles of A single stator are composed of three groups of piezoelectric layers, which are represented as A, B, and C respectively, and each group of piezoelectric layers realizes different modes of motion. Under the action of group A, the stator is bent up and down along the positive and negative semi-axes of the Z axis. Group B realizes telescopic movement along the Y-axis of the stator direction, and Group C generates bending vibration along the positive and negative semi-axes of the X-axis. The flange junction structure is added between each group of piezoelectric plates, the main purpose is to adjust the size and achieve better degeneracy of the three mode frequencies. At the same time, the increase of flange improves the overall length of the stator, which helps to improve the displacement. The polarized output displacement of the stack piezoelectric ceramics can be understood as the superposition of the output displacement of each piezoelectric ceramic plate.

(4) $$E=\frac{u}{d}$$

(5) $$n=\frac{l}{d}$$

(6) $$\Delta_{t}=d_{33}\times u$$

(7) $$\Delta_{l}=n\times \Delta_{t}=n\times d_{33}\times u$$

where u the excitation voltage at both ends of piezoelectric ceramics, E electric field strength, d single piezoelectric ceramic thickness, l the total length of stacked piezoelectric ceramic, n the number of layers of piezoelectric ceramics, $\Delta_{t}$ output displacement of the single piezoelectric ceramic, $\Delta_{l}$ total displacement of stacked ceramic sheets, $d_{33}$ piezoelectric constant.

The top of the stator driving foot is cut by arc, and the arc surface just fits with the spherical rotor so that it can better clamp the rotor to do multi-degree of freedom movement. The specific operation mode will be introduced in the third section. The supporting structure is fixed around the ball in contact with the lower surface of the ball. It mainly plays two roles, one is to fix the height of the ball in the equatorial plane and the stator axis tangent position, and the other is to design the contact part of the spherical ball as far as possible to reduce the friction between the ball and the rotor, the ball and the rotor contact area is small, approximate to the point contact, so it can avoid too large friction and affect the output rotation of the motor.

### 2.2. Working Principle

**Figure 5:**
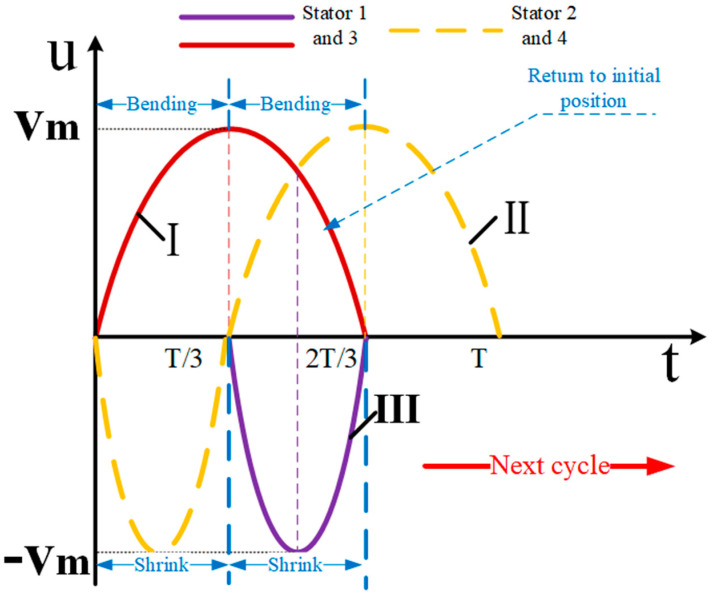
Signal 1.

The operation of the proposed elephant trunk motor is based on the flexural, longitudinal-flexural complex motion of the four driving feet in the stator resonance state. Both longitudinal vibration and bending vibration are second-order modes. The piezoelectric ceramic plate adopts the mode with a higher electromechanical coupling rate. The stator piezoelectric ceramics of the proposed motor are driven by sinusoidal signals with the same amplitude and frequency. There are mainly two kinds of driving signals, and different degrees of freedom can be realized under the driving of these two signals. As shown in Figure 5, the first sinusoidal driving signal is given, under which two different degrees of freedom can be realized.

**Figure 6:**
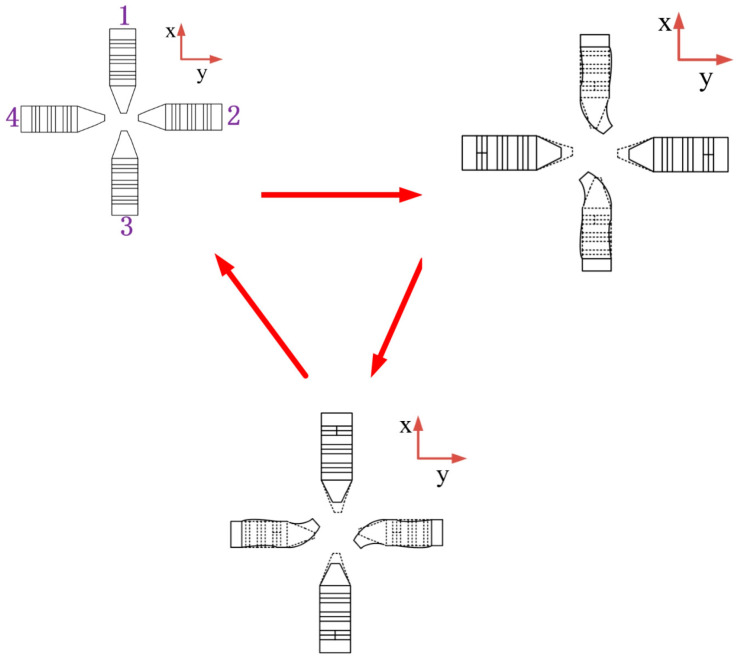
Motion process of mode 1.

Mode 1: First, label the four stators clockwise as 1,2,3,4. In the process of moving around the Z-axis, two diagonally matched stators are adopted. The excitation mode shown in Figure 5 is selected for signals, and the process is shown in Figure 6.

When t = 0, the four stators are all in a static state without any change.

When t = 0~T/3, the signal I is applied to the C piezoelectric ceramics of the 1 and 3 stators at the same time, which makes the two stators’ driving feet produce bending vibration displacement in the same direction. At the same time, signal II is applied to the B piezoelectric ceramics of the 2 and 4 stators, which makes the stators do positive displacement and negative displacement along the X axis, respectively. At this point, the No. 1 stator and No. 3 stator will clamp the spherical rotor and rotate. When t = t/3, because the voltage of signal Ⅰ reaches the peak value, the bending displacement of No. 1 and No. 3 stators also reaches the maximum deformation. At this point, the signal Ⅱ returns to its original point, and the signals II and IV return to their original invisible state.

When t = t/3~T2/3, the signal Ⅱ is applied to the C piezoelectric ceramics of the No. 2 stator and No. 4 stator at the same time, so that the two stator driving feet produce bending vibration displacement in the same direction. At this time, the signal Ⅰ continues to be applied to the c ceramic plates of the 1 and 3 stators, while the signal Ⅲ is applied to the B ceramic plates of the 1 and 3 stators. The purpose of this is to keep the 1 and 3 stators out of contact with the spherical rotor, so the motion of the two stators is to shrink while recovering the deformation. When t = 2T/3, the No. 2 and No. 4 stators reach their ultimate displacement and no longer drive the rotor to rotate, so the No. 1 and No. 3 stators also return to their initial positions.

When t = 2T/3~T, the hold signal Ⅱ is still applied to the stators of signals II and IV, and when the signal reaches the origin, the stators return to their original state.

The above is a detailed description of a complete cycle of mode 1. When the signal is applied to the stator continuously, the rotor can realize continuous operation without any idle waiting time. If you go counterclockwise about the Z axis you just apply the opposite signal as described above.

**Figure 7:**
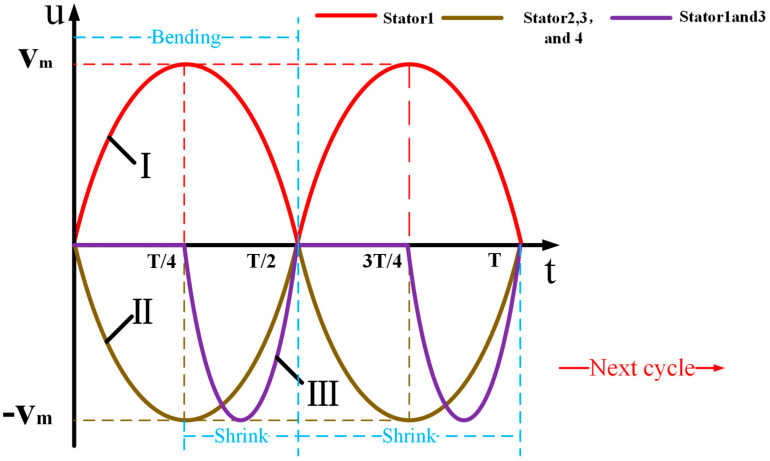
Signal two.

**Figure 8:**
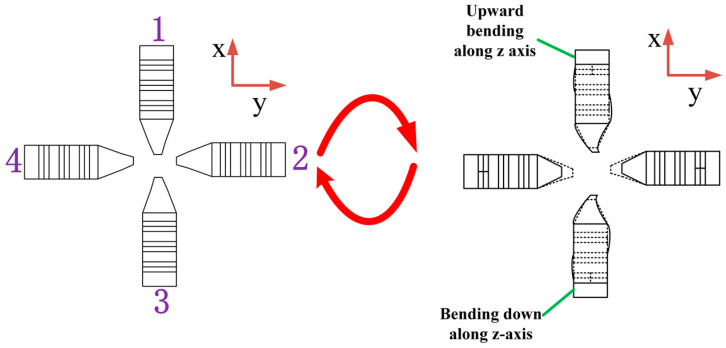
Motion process of mode 2.

Mode 2: Movement around the Y-axis. The movement in this mode is relatively simple and only requires two diagonal stators to hold the rotor for movement. The signal selection is shown in Figure 7, in which the two bending vibration change meters of the stator are integrated into one cycle. The motion process is shown in Figure 8.

When t = 0, the four stators are all in a static state without any change

When t = 0~T/4, and by applying signal Ⅰ to Group A piezoelectric ceramic of No. 1 stator and signal Ⅱ to Group A piezoelectric ceramic of No. 3 stator, the two stator driving feet produce bending vibration displacement in opposite direction. At the same time, signal Ⅱ has also been applied to Group B piezoelectric ceramics with no. 2 and No. 4 stators, and the stator contractions do not touch the rotor. The No. 1 and No. 3 stators will rotate the spherical rotor around the Y-axis for displacement. When t = T/4, the No. 1 stator and No. 3 stator will no longer drive the rotor to rotate when they reach the limit displacement, and the No. 2 stator and No. 4 stator will reach the maximum shrinkage deformation.

When t = T/4~T/2, the signal applied by the No. 2 stator and No. 4 stator is unchanged, and the signal III is applied to the B ceramic plate of the No. 1.3 stator at the time of T/4. At this time, the No. 1 stator and No.3 stator recover their deformation while shrinking and are not in contact with the rotor. The No. 2 and No. 4 stators will also be restored to their original state.

When t = T/2, the four stators return to the initial state.

In the second mode, when the rotor moves forward around the Y axis, there is a neutral waiting time, and the waiting time is when No. 1 and No. 3 stators can be restored to the initial state before the spherical rotor can be mechanically driven to operate. Therefore, follow-up work is needed to explore how to make continuous operations around the Y-axis. If you are going in the opposite direction about the y-axis, you just have to apply the opposite signal. If you are moving about the X-axis, you only need to change the stator, and the signal is applied in the same way as above.

**Figure 9:**
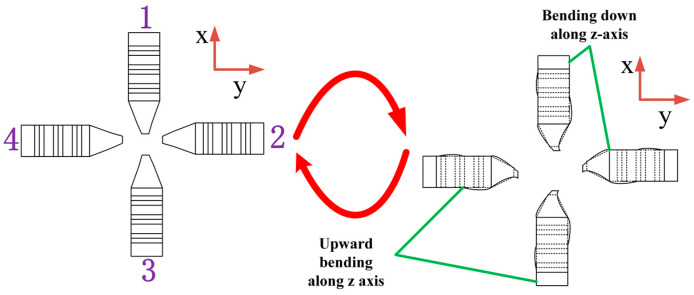
Motion process of mode 3.

Mode 3: Motion is obtained perpendicular to the diagonal plane. This mode requires four stators to work together for movement. The excitation mode in Figure 7 is selected as the signal, and the motion mode is shown in Figure 9.

When t = 0~T/4, signal I has applied to Group A ceramic plates of No. 3 and No. 4 stators, and signal II was applied to Group A ceramic plates of No. 1 and No. 2 stators. This will push the rotor to rotate perpendicular to the diagonal plane.

When t = T/4, the amplitudes of all four stators reach their maximum.

When t = T/4~T/2, at this time, the signal III is added to the process of movement, and the III signal is applied to all the stator group b ceramic pieces. The four stators shrink and reset with flexural vibration. When t = t/2, all stators return to the initial position.

When t = T/2~T Start the next cycle.

A total of four diagonal planes can be achieved by simply applying the signal to different stators.

For all the degrees of freedom presented by the elephant trunk ultrasonic motor designed above, when compared with mode 1, mode 2 and mode 3 do not realize continuous operation, and there is a T/4 cycle gap between them from the next clamping operation. Therefore, this is also a problem that needs to be solved and improved in the future.

## 3. Stator-Rotor Dynamics Modeling

In this paper, according to the principle of the designed elephant trunk ultrasonic motor with multiple degrees of freedom, a theoretical analysis of the fixed rotor contact is carried out, and the fixed rotor contact is described. In the actual operation of the motor, normal force and tangential force as well as normal displacement and tangential displacement will be generated. The type of variable of the driving foot is different at different times, which can be equivalent to the change of displacement. Therefore, the spring-mass-damping system is used here to establish the relationship between displacement and time to describe its dynamic model [19]. The stator rear end cover of the designed motor relies on nuts to exert pre-pressure on the whole stator so that it is fully in contact with the rotor, the base plays a fixed role, and the whole motor is integrated into a whole through bolts. In the equivalent model, the application of pre-pressure and the part of the driving foot in contact with the rotor are assumed to be an elastomer, and displacement is only output at one end of the driving foot.

**Figure 10:**
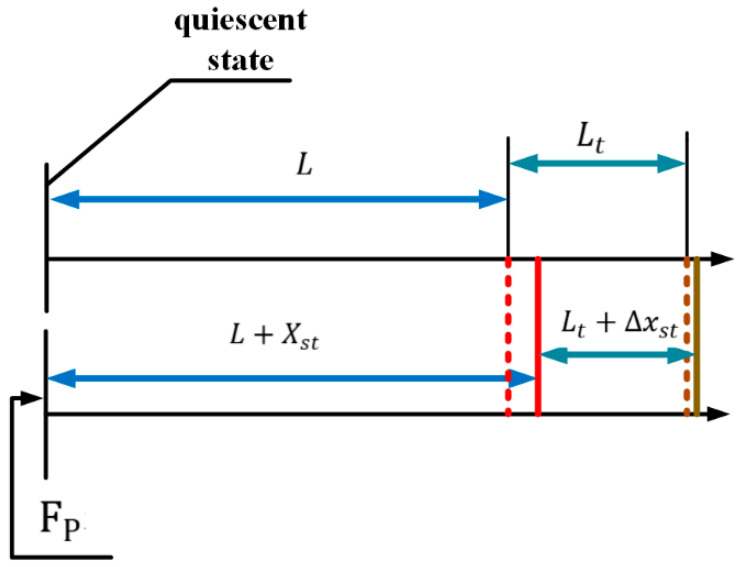
Shows the static displacement of the pre-pressure under the number line.

First, the normal force and tangential force are analyzed. The normal force is expressed as follows:

(8) $$F_{N}=\left\{ \begin{array}{l} F_{p}+k_{y}\left(x_{d}-x_{st}\right),when\;F_{p}+k_{y}\left(x_{d}-x_{st}\right)>0\\ 0,when\;F_{p}+k_{y}\left(x_{d}-x_{st}\right)>0 \end{array}\right.$$

where, $F_{P}$ is the applied pre-pressure, $K_{y}$ is the axial elastic coefficient, and $x_{d}$ is the total length of the stator’s modal extension at 43.027 kHz, and is expressed as

(9) $$x_{d}=u_{l}\left(\omega_{u}\left(L\right)-\omega_{u}\left(0\right)\right)$$

where, $\omega_{u}\left(x\right)$ is the eigenfunction of the Bernoulli-Euler beam, corresponding to the second-order longitudinal vibration mode, and $u_{l}$ is the displacement along the axial direction. In the formula, $x_{st}$ is the static displacement under the action of preloading force. When the pre-pressure is applied, the piezoelectric body will have a small deformation, so that the compression spring, that is, the foot part of the deformation, can be obtained by the following formula:

(10) $$x_{st}=\frac{\gamma_{n}F_{P}}{k_{pzl}}\left(\omega_{u}\left(l\right)-\omega_{u}\left(0\right)\right)$$

where, $\gamma_{n}$ is the load coefficient and $K_{pzl}$ is the stiffness of the longitudinal vibration mode. $x_{st}$ is represented by the number line, as shown in Figure 10.

(11) $$\Delta x_{st}=-\frac{F_{P}}{K_{P}}$$

When a signal is applied to the group B piezoelectric plate, the axial force will be generated. The axial force is defined as dynamic deformation, which is the same as that when pre-pressure is applied. For both the piezoelectric body and driving foot, deformation displacement increases.

(12) $$F_{N}=k_{p}\times \Delta x_{d}$$

When, $F_{P}+k_{y}\left(x_{d}-x_{st}\right)=0$, $x_{d}=x_{st}-\frac{F_{P}}{k_{p}}$ thus prestressing stress affects the axial force and displacement of the rotor, the contact between Therefore, proper pre-pressure can ensure reasonable contact of the rotor and reduce the loss caused by friction. When $x_{d}<x_{st}-\frac{F_{P}}{k_{p}}$ the fixed rotor will not be in contact.

The tangential force is generated by the sliding friction between the driving foot and the contact surface of the rotor when the piezoelectric ceramic is excited, and the bending vibration occurs. Tangential force is expressed as follows:

(13) $$F_{T}=sign\left(\overset{•}{v_{st}}-\overset{•}{v_{t}}\right)\mu F_{N}$$

where

(14) $$sign\left(x\right)=\{\begin{array}{c} 1,x>0\\ 0,x=0\\ -1,x<0 \end{array}$$

According to the balance of forces, the dynamic equation of the rotor can be obtained as follows:

(15) $$m_{2}\overset{•}{v_{t}}+c_{t}v_{t}=F_{t}+F_{f}-F_{load}$$

The $m_{2}$ for rotor quality, $v_{st}$ driven sufficiently places a point of tangential velocity, $v_{t}$ for the rotor speed, $c_{t}$ for contact interface friction coefficient. When measuring the motor performance, the speed of electrons under load and no load is usually studied. Therefore, the load $F_{load}$ is added to this formula. If $F_{load}=0$, the no-load speed will be obtained. $F_{f}$ represents a series of frictions that occur in the system, including the effects of gravity and normal alignment. The friction force between the rotor and the driving foot can be expressed as follows:

(16) $$F_{f}=\{\begin{array}{c} -F_{t},\left|F_{t}\right|<\left|F_{f\max}\right|\\ F_{f\max},\left|F_{t}\right|\geq \left|F_{f\max}\right| \end{array}$$

With

(17) $$F_{f\max}=sign\left(-v_{t}\right)\delta \left(F_{n}+m_{2}g\right)$$

where δ is the friction coefficient and g is the gravitational acceleration. From a design point of view, the friction should be as low as possible but only if it is greater than the max to keep the rotor running.

**Figure 11:**
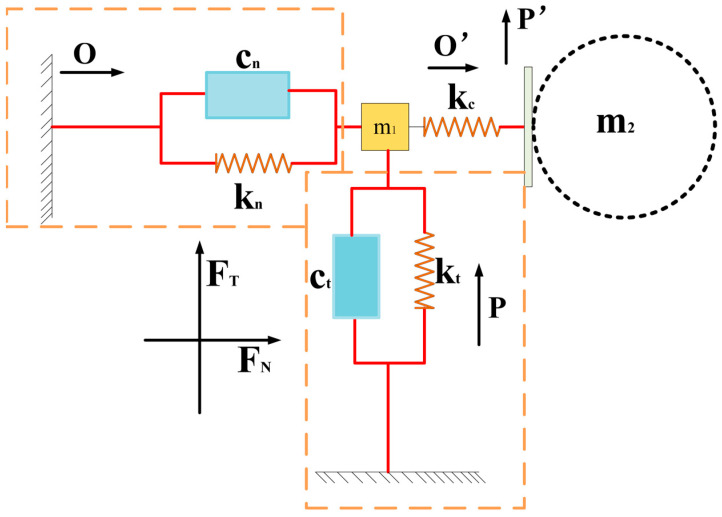
Dynamics model of two degrees of freedom.

The overall dynamic model of the motor is shown in Figure 11. The operation of the motor is matched by two stators, so the system model of two degrees of freedom is established. The whole system includes spring, damping, stator mass element, and spherical rotor section. The driving foot drives the rotor in a circular motion, and the displacement is very small. However, the arc trajectory can be differentiated into several small displacements, which are approximately equivalent to linear motion.

The typical structure of a piezoelectric actuator is composed of a preload mechanism, a base, and a stator. The stator is the key driving component, which is composed of a piezoelectric stack and a driving mechanism. The whole stator part will deform and restore the deformation under the action of the piezoelectric effect of piezoelectric ceramics, so this process can be equivalent to the spring system. Considering the damping coefficient of the material itself, the influence of this aspect is taken into account in the established model. In the actual process, the piezoelectric part will cause deformation, and the driving foot will also cause small deformation due to the contact with the rotor, namely, $k_{c}$ in Figure 11. However, qualitative analysis is not made here because the deformation is very small. From the description of the previous chapter, it can be seen that the stator can realize both vertical and tangential motion, so the two degrees of freedom correspond to these two modes of motion respectively. The stator designed in this paper mainly drives the rotor to rotate by its tangential force. When the stator moves tangentially, $k_{t}$ deforms and generates tangential force. A tangential force acts on the rotor and drives the rotor to do micro-displacement movement by friction.

$m_{1}$ is defined as the mass of the stator, $c_{n}$ and $c_{t}$ are denoted as tangential and axial damping coefficients respectively, $k_{t}$ and are $k_{n}$ denoted as tangential and axial spring coefficients, tangential input displacement and normal input displacement are denoted by O and P respectively, output displacement is denoted by $\overset{•}{O}$ and $\overset{•}{P}$, and the number is denoted as the stator label. Based on the consideration of tangential force and normal force generated in stator bending vibration, the motion equation of the driving foot is as follows:

(18) $$m_{1f}\overset{\cdot \cdot }{O^{’}}\left(t\right)+c_{t}\overset{\cdot }{O^{’}}\left(t\right)+k_{t}O^{’}\left(t\right)=F_{x}\left(t\right)-F_{t}\left(t\right)m-\overset{\cdot \cdot }{P^{’}\left(t\right)}+c_{n}\overset{\cdot }{P^{’}}\left(t\right)+k_{n}P^{’}\left(t\right)=F_{y}\left(t\right)-F_{n}\left(t\right)$$

Both $F_{x}\left(t\right)$ $F_{y}\left(t\right)$ are determined by tangential and normal input displacements.

(19) $$F_{x}\left(t\right)=c_{t}\left(t\right)\overset{\cdot }{O}\left(t\right)+k_{t}O\left(t\right)$$

(20) $$F_{y}\left(t\right)=c_{n}\overset{\cdot }{P}\left(t\right)+k_{n}P\left(t\right)$$

It is known from the piezoelectric equation that the force on the piezoelectric ceramic is proportional to the amplitude of the applied voltage, and it is also related to the $d_{33}$ coefficient. Therefore, the input displacement can be expressed by the following formula:

(21) O(t)=∂Usin(2πft)P(t)=δUsin(2πft+φ)

Wherein, ∂ and δ are electromechanical coupling coefficients, U is voltage amplitude and f is resonance frequency.

**Figure 12:**
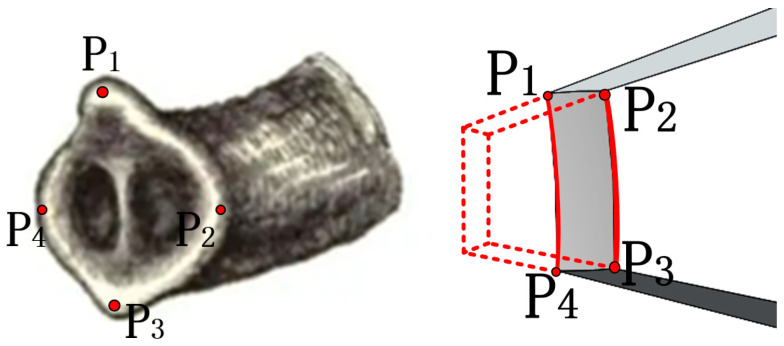
Stator section processing.

**Figure 13:**
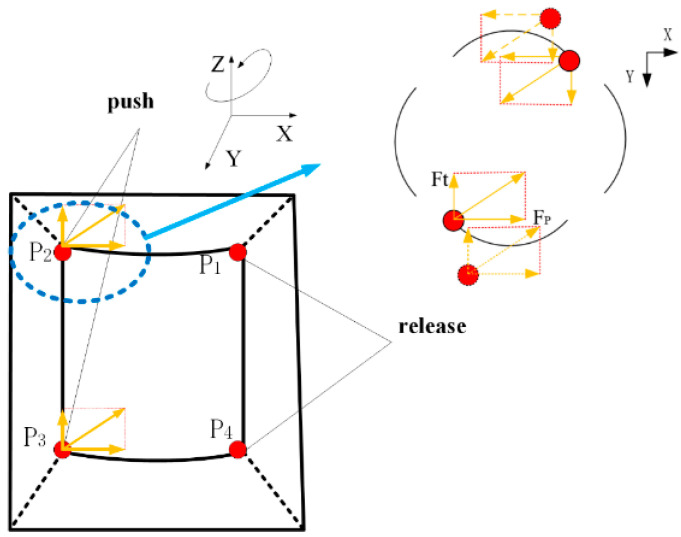
Force analysis of the contact between the driving foot and the rotor.

As mentioned above, in order to better fit the rotor, the driving foot is specially planned, as shown in Figure 12. The elephant trunk is designed. The four fulcrum points $P_{1}P_{2}P_{3}P_{4}$ do not exert force on the spherical rotor together. When the rotor has flexural vibration displacement, two fulcrum points will be separated from the rotor, and the remaining two fulcrum points will push forward the movement, as shown in Figure 13. When the rotor rotates around the Z axis, the two points of P1P4 release and do not contact the rotor, and the remaining two points will push it to rotate. In fact, in the process of rotor rotation, the force on the rotor is gradually transferred from the surface to the line at the last point, so the force on the rotor is enough for it to rotate. However, all the forces in this paper are summed up into one point for analysis. According to the above, the rotor is first subjected to $F_{p}$, followed by the tangential force $F_{t}$ generated during bending vibration. The two forces are synthesized, as shown in the Figure. Through observation, it can be found that the resultant force is always tangent to the sphere so that the spherical rotor can receive the maximum output tangential force to realize the selection. In fact, with the change of time in stator bending vibration, the direction of the force is constantly changing, and the contact area between the fulcrum and the ball is also constantly changing, but it is always along the tangential direction of the ball to promote the rotor movement.

## 4. Driver Model of Electromagnetic-Piezoelectric Hybrid Drive Motor

### 4.1. Modal Analysis

**Figure 14:**
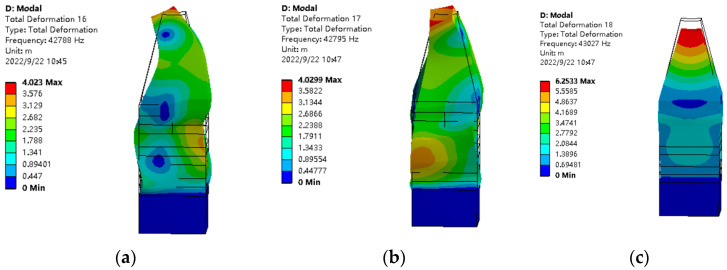
Vibration modes under three different vibration modes (**a**) 42.788 kHz (**b**) 42.795 kHz (**c**) 43.027 kHz.

The modal analysis and harmonic response analysis of the whole stator were carried out using ANSYS Workbench software, and the stator parameters were constantly adjusted according to the modal analysis results to achieve frequency degeneracy. The polarization and parameter setting of piezoelectric ceramics were processed in Workbench software. The three working modes are shown in Figure 14, and the frequencies are 42.788 kHz, 42.795 kHz, and 43.027 kHz, respectively. The difference between these three characteristic frequencies is 0.007 kHz and 0.268 kHz respectively, and the difference is less than 0.6% of the stator resonant frequency, which indicates that the three vibration modes achieve good degeneracies.

### 4.2. Harmonic Response

**Figure 15:**
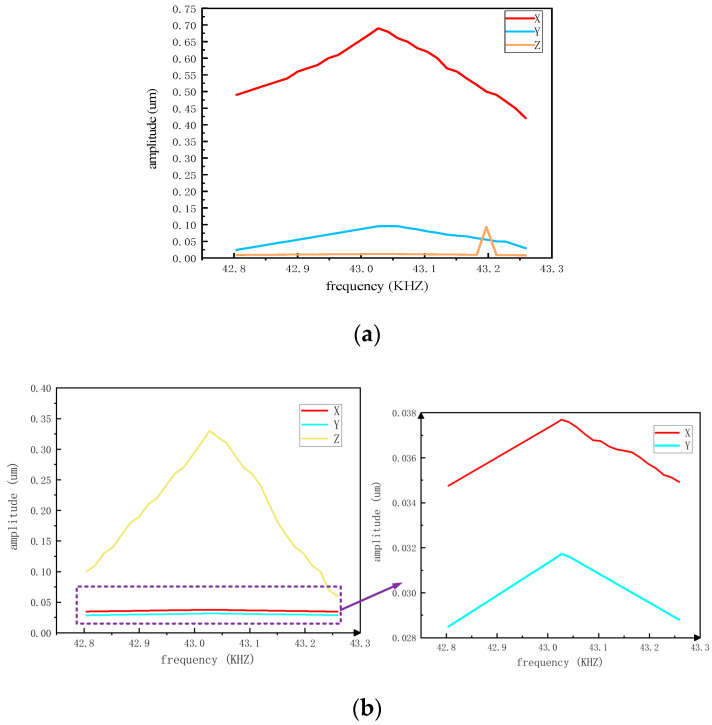

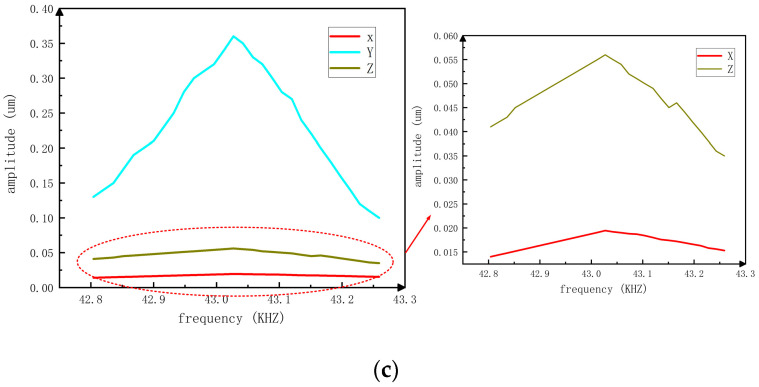
(**a**) Group A piezoelectric ceramics, (**b**) Group B piezoelectric ceramics, (**c**) Group C piezoelectric ceramics.

In the process of harmonic response analysis of the stator, an AC voltage of 400 V was applied to three groups of piezoelectric ceramic plates respectively to test the resonant frequency and displacement output along the X, Y, and Z axes under the action of each group of separate ceramic plates. As shown in Figure 15, the resonant frequency of each group of ceramic sheet states is close to 43.027 kHz. Through the comparison of the amplitudes, it is observed that when each group of ceramic plates acts alone, the displacement in their own fixed motion direction is the maximum, while the output displacement in the other axes is orders of magnitude larger than the maximum. It can be concluded that the stator has no current amplitude deviation.

### 4.3. Experimental Test

**Figure 16:**
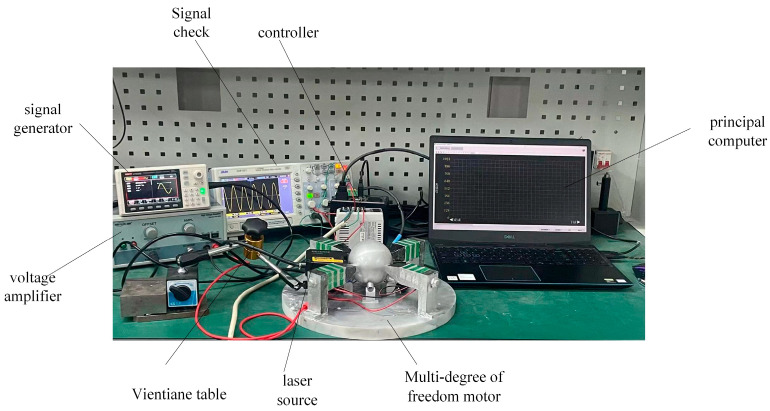
An experimental platform of elephant-trunk multi-degree of freedom motor.

The experimental test platform established is shown in Figure 16, which is used to test the speed that the rotor can achieve and the load capacity of the motor under different excitation voltages and frequencies. First, by means of a signal generator U8793 (Japanese HIOKI, Shanghai, China), the generator can send any waveform and voltage signal of any frequency according to the need. After debugging the required sinusoidal voltage signal, the signal is amplified by the voltage amplifier HA-820 (Pintech, Xi’an, China) according to the calculated ratio and applied to the lamination. After adjusting the position of each part, the laser sensor LK-H008 (Japanese KEYENCE, Shanghai, China) is secured using the Vientiane base to align its laser beam with the slide rail. The sensor head type is light spot type, and the repetition accuracy can reach 0.005 um. The displacement data obtained by the data collector is uploaded to a PC for sorting and analysis.

#### 4.3.1. Output Displacement under Different Driving Voltages

**Figure 17:**
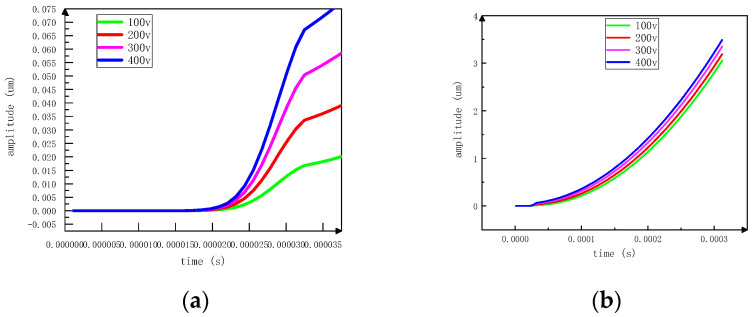
Displacement changes at different voltages (**a**) time-displacement relation curve of one cycle (**b**) superposition of 60 periodic displacements.

At the resonant frequency of 43.027 kHz, voltage signals of different amplitudes are applied, and the displacement data of the rotor are observed and stored in real-time by a laser vibrometer. Motion mode 1 mentioned in the third chapter is tested experimentally. The displacement in a period is shown in Figure 17a. The displacement is proportional to the time in a period. When the stator deformation reaches the maximum, the displacement will not change. The displacement is 0.015 um at 100 V voltage and 0.075 um at 400 V voltage. At the same time, it can be observed that the step displacement is not obvious at 100 V voltage. This is because the driving effect of the piezoelectric actuator on the rotor is based on the superposition of multiple periodic micro-displacements, so the movement is slow at this time. The step displacement begins to increase with the increase of voltage, but the displacement curve is relatively smooth. Since the signal applied to the ceramic is a continuous voltage signal, the rotor rotates continuously in one cycle. It can be proved that the driving effect above 100 V is stable. Then the output displacement of 60 cycles is calculated, as shown in Figure 17 b. The displacement curve is closer to the exponential function and shows an increasing trend. When the rotor is driven, the stator will have a certain response time. At this frequency, the point delay time is 0.0185 ms.

From the experimental results, it can be seen that the increase in displacement depends on the change in voltage, which can be explained by Formula (21). The given publicity indicates that the output displacement is a sin function of time T, so the displacement will increase with time at the same voltage. At the same time, the higher the voltage, the greater the corresponding output displacement.

#### 4.3.2. Test the Relationship between Rotor Speed and Voltage Frequency

**Figure 18:**
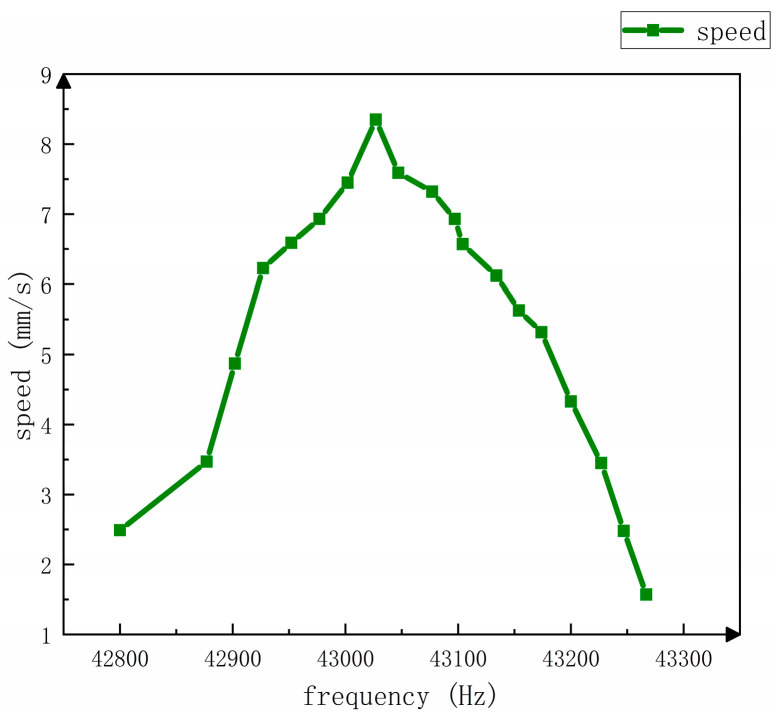
Relationship between speed and frequency.

**Figure 19:**
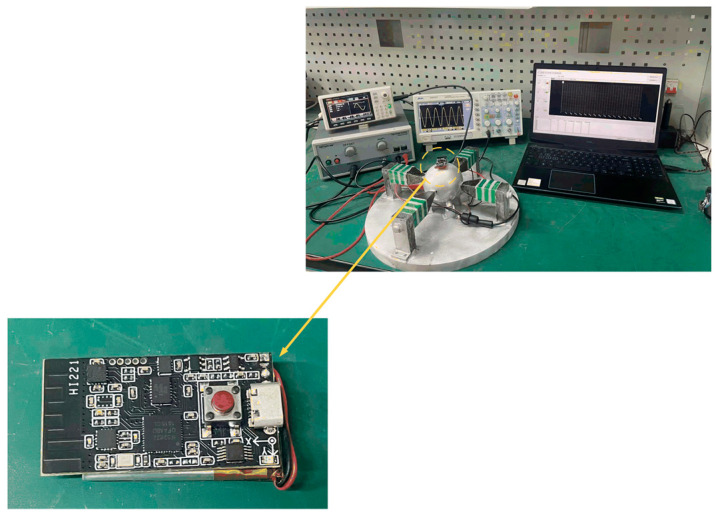
Speed test platform.

**Figure 20:**
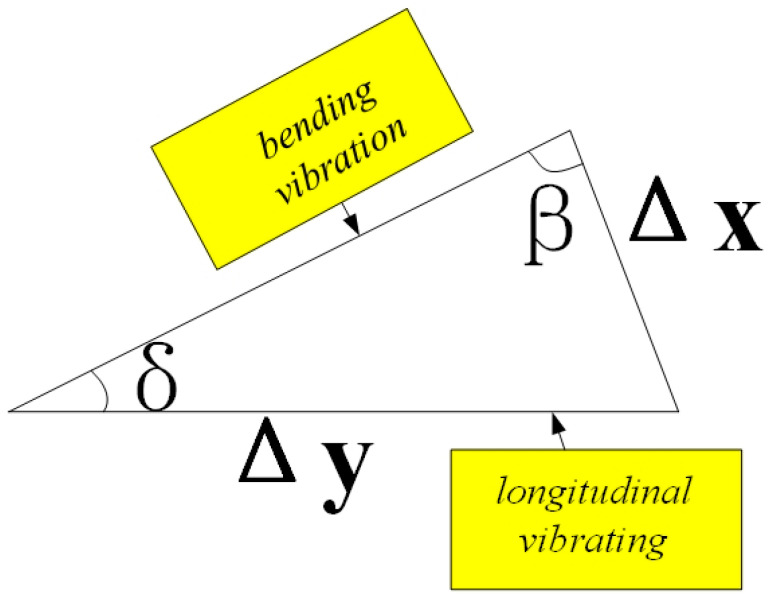
The relationship between tangential displacement and axial displacement.

The voltage is set at 400 v, and the relationship between rotor speed and frequency is shown in Figure 18. Figure 19 shows the experimental test platform. When the frequency is 42.877 kHz, the rotor speed rises slowly. When the frequency is greater than 42.877 kHz, the rotor speed increases and speeds up obviously. When the frequency is greater than 42.977 kHz, the rotor speed slows down again. The maximum rotor speed is 8.56 mm/s. The following qualitative analysis can be completed in this experiment, as shown in Figure 20, Δx which is defined as tangential displacement and Δy is defined as axial displacement. When the stator is bent and deformed, an angle is formed with the stator that only has axial displacement, which is defined as δ. Then the relationship between the two displacements is:

(22) $$\Delta x\left(t\right)=\frac{\sin\delta \left(t\right)}{\sin\beta \left(t\right)}\Delta y\left(t\right)$$

From the above formula, it is known that the tangential displacement is closely related to the axial displacement at a given time $t_{1}$. The axial displacement is expressed as:

(23) $$\Delta y\left(t\right)=D_{1}\sin\left(2\pi ft+\varphi \right)$$

$D_{1}$ Longitudinal amplitude determined by the excitation condition, φ Initial phase of longitudinal vibration [19].

Therefore, at the time $t_{1}$, the formula is rewritten as:

(24) $$\Delta x\left(t_{1}\right)=\mu \sin\left(2\pi ft_{1}+\varphi \right)$$

The visible frequency will affect the output of the tangential displacement, so the measured curve is also similar to the sin function curve.

#### 4.3.3. Motor Load Capacity Test

**Figure 21:**
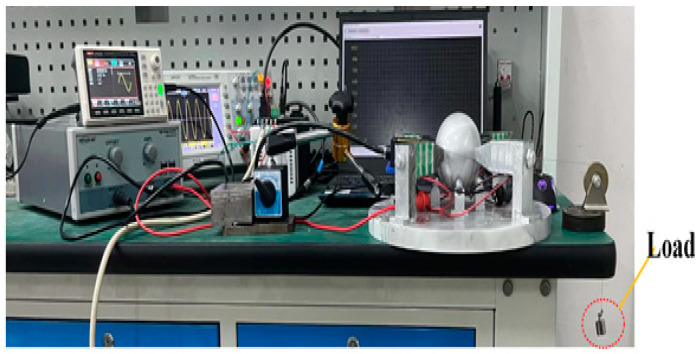
Load test platform.

**Figure 22:**
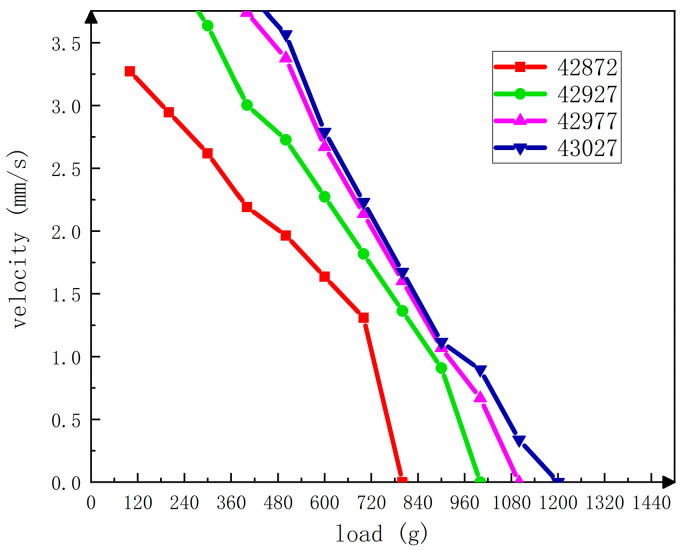
Load capacity test of the motor.

Finally, the load capacity of the motor was analyzed, and the sheaves—ropes—weights load test system was established, as shown in Figure 21. The fine wire was wrapped around the spherical rotor, and weights were added to the other end. Four frequencies were selected respectively for load analysis. The load is selected to be in the order of 100–1200 g and increased according to 100 g. It can be seen from Figure 22 that 43.027 kHz can carry 1200 g of load, and the load capacity of the motor is further improved with the increase in frequency. From the analysis of the previous section, it can be known that the initial speed of different resonant frequencies is different. Due to the limited bearing capacity of the motor under different conditions, the speed will gradually decrease with the increase in weight, which is in line with common sense. At the same time, due to the greater weight, the friction between the fine line and the pulley will also increase accordingly, which will lead to a faster downward trend in speed.

## 5. Conclusions

The elephant trunk piezoelectric stator proposed in this paper improves the utilization rate of the stator and drives the spherical rotor to realize multi-freedom motion, which changes the previous single-freedom and linear motion modes. Firstly, the driving principle of the driving foot and the overall dynamic model of the motor is analyzed through the designed model mechanism. Through the overall design of the stator structure, the longitudinal-bending—longitudinal combined vibration is realized in the driving foot. The three modes all reach the resonant frequency at 43.027 kHz, which proves the rationality of the stator design. The output characteristics of the motor are tested by the experimental platform. As shown by experimental data the motor speed can reach 8.56 mm/s and the deflection displacement of 0.69 um under 43.027 kHz and 400 V drive. At the same time, it can drive a 1200 g load under the set driving conditions.

The proposed motor has better flexibility compared to linear guides because of the spherical structure of the rotor. For some places requiring precise travel, for example, cell injection in the medical industry, robot joints in the engineering industry need flexible rotation and also need a certain load-bearing capacity. The designed motor is a combination of longitudinal vibration and bending vibration, which achieves a certain load capacity while realizing multiple degrees of freedom. However, there is still room for improvement.

Although this paper puts forward a new idea in the aspect of multi-degree of freedom, in the previous introduction of structural principle, mode 2 and mode 3 cannot achieve continuous work, so the follow-up work can try to continue to improve from the design of working principle or the optimization of stator structure. Secondly, the practical application of the motor is also a major focus of the follow-up research work. Combined with the motor and the actual working conditions, the overall structure of the motor is continuously improved and optimized in terms of displacement, speed, and bearing capacity.

## Figures and Tables

**Table 1:** Stator Materials.

Materials	Density	Young’s Modulus	Poisson’s Ratio
PZT-5	7500	7.8 × 10^10^	0.35
Al	2700	7.8 × 10^10^	0.33
Structural steel	7800	2.02 × 10^11^	0.30

**Table 2:** Motor Parameters.

Parameter	$l_{1}$	$l_{2}$	$l_{3}$	$l_{4}$	$l_{5}$	$l_{6}$
Numerical value (mm)	6	41	5	8	16.9	30
Parameter	$d_{1}$	$d_{2}$	$d_{3}$	$d_{4}$	$R_{1}$	$R_{2}$
Numerical value (mm)	30	5	32	20	6	35

## Data Availability

The data that support the findings of this study are available from the corresponding author upon reasonable request.
